# Lentiviral transduction of Tar Decoy and CCR5 ribozyme into CD34+ progenitor cells and derivation of HIV-1 resistant T cells and macrophages

**DOI:** 10.1186/1742-6405-1-2

**Published:** 2004-12-17

**Authors:** Akhil Banerjea, Ming-Jie Li, Leila Remling, John Rossi, Ramesh Akkina

**Affiliations:** 1 Dept. Microbiology, Immunology and Pathology, Colorado State University, Fort Collins, Colorado 80523, USA; 2 Division of Molecular Biology, Beckman Research Institute of the City of Hope, 1450 East Duarte Road, Duarte, California, 91010, USA

**Keywords:** AIDS gene therapy, HIV tar decoy, CCR5 ribozyme, SCID-hu mice, Lentiviral vectors, HIV aptamers, CD34 cells

## Abstract

**Background:**

RNA based antiviral approaches against HIV-1 are among the most promising for long-term gene therapy. These include ribozymes, aptamers (decoys), and small interfering RNAs (siRNAs). Lentiviral vectors are ideal for transduction of such inhibitory RNAs into hematopoietic stem cells due to their ability to transduce non-dividing cells and their relative refractiveness to gene silencing. The objective of this study is to introduce an HIV-1 Tar aptamer either alone or in combination with an anti-CCR5 ribozyme into CD34+ hematopoietic progenitor cells via an HIV-based lentiviral vector to derive viral resistant progeny T cells and macrophages.

**Results:**

High efficiency and sustained gene transfer into CD34+ cells were achieved with lentiviral vector constructs harboring either Tar decoy or Tar decoy in combination with CCR5 ribozyme. Cells transduced with these constructs differentiated normally into T-lymphocytes *in vivo *in thy/liv grafts of SCID-hu mice, and into macrophages *in vitro *in the presence of appropriate growth factors. When challenged *in vitro*, the differentiated T lymphocytes and macrophages showed marked resistance against HIV-1 infection.

**Conclusions:**

Viral resistant transgenic T cells and macrophages that express HIV-1 Tar aptamer either alone or in combination with an anti-CCR5 ribozyme could be obtained by lentiviral gene transduction of CD34+ progenitor cells. These results showed for the first time that expression of these anti-HIV-1 transgenes in combination do not interfere with normal thymopoiesis and thus have set the stage for their application in stem cell based gene therapy for HIV/AIDS.

## Background

Human T lymphocytes and macrophages are the major host cells for HIV-1 replication. The initial infection is established by macrophage tropic viruses (R5) that use the chemokine receptor CCR5 and CD4 to gain entry into a susceptible host cell. During the later stages of the disease, T-cell tropic viruses (X4) that use CXCR4 as a coreceptor predominate [1,2]. Since HIV-1 coreceptors play a key role during the early viral-cell interactions, they are attractive targets for many antiviral approaches. A 32-base pair deletion in the CCR5 gene found in a segment of the normal European and North-American population rendered their macrophages resistant to infection by R5-tropic HIV-1 [3]. Since these individuals lacking a functional CCR5 are apparently normal, this gene has been targeted by many investigators to confer HIV-1 resistance. Using MuLV vectors for gene delivery, ribozymes or DNA-enzymes targeted against CCR5 were previously shown to inhibit HIV-1 entry both *in vitro *and *in vivo *in a SCID-hu mouse model [4-6]. Efficacy of siRNAs in down regulating the CCR5 coreceptor and thereby preventing HIV-1 entry was also described recently [7,8].

The regulatory proteins Tat and Rev encoded by the viral genome are indispensable for HIV-1 gene expression and replication. The Tat protein interacts with the bulged RNA region of the transactivation response element (Tar), present at the 5'-end of all HIV-1 transcripts [1]. In the absence of Tat, only short ineffective transcripts are generated. Tat is also known to interact with cellular factors like cyclin T1 and cyclin dependent kinase (Cdk9). Since Tat plays a critical role in virus replication, it is an ideal target. Effective inhibition of HIV-1 replication was shown earlier by the use of Tar specific RNA decoys and ribozymes [9-11]. Moreover, siRNAs directed against Tat were also found to be highly potent in inhibiting HIV-1 replication in cultured cell lines and in PBMCs [12,13]. However, development of viral resistance and generation of escape mutants are possible obstacles for long range efficacy of these constructs as exemplified by the recent findings of Boden et al [14]. These obstacles can be overcome by the use of combinatorial constructs against multiple targets in the viral genome as well as cellular targets that assist in viral infection and replication.

CD34+ hematopoietic progenitor stem cells (HPCs) are ideal targets for transducing anti-HIV genes as they give rise to both T cells and macrophages which are the main viral targets. Most of the previous work with anti-HIV ribozymes and RNA decoys employed conventional MuLV derived retroviral vectors to transduce these cells [5,6,11]. However, the efficiency of gene transduction by these vectors is relatively low as they are unable to transduce non-dividing cells. In addition, the transgenes carried by these vectors are prone to gene silencing during the differentiation of end stage cells such as T cells and macrophages [15]. On the contrary, lentiviral vectors appear not to have these limitations [16,17]. Based on these advantages, we used the new generation lentiviral vectors to achieve high level gene transfer and sustained gene expression. A ribozyme against CCR5 and a Tar aptamer decoy were previously shown to inhibit HIV-1 in transduced cells [6,18,19]. In previous *in vivo *studies, MuLV based conventional retroviral vectors were used. In addition, it is not known if Tar decoy is effective in *in vivo *differentiated thymocytes. In these studies, our goal is to determine the utility of these constructs when introduced into human CD34+ progenitor cells via an HIV-1 based lentiviral vector to derive HIV resistant differentiated target cells both *in vitro *and *in vivo*. Using an *in vitro *cell differentiation system and *in vivo *SCID-hu mouse model, we show that expression of Tar decoy alone or in combination with an anti-CCR5 ribozyme has no adverse effect on lineage specific differentiation of CD34+ cells into macrophages and T lymphocytes. We also show that the transgenic cells display resistance to HIV-1 challenge.

## Results

### High efficiency transduction of CD34+ cells with lentiviral constructs

**Figure 1:**
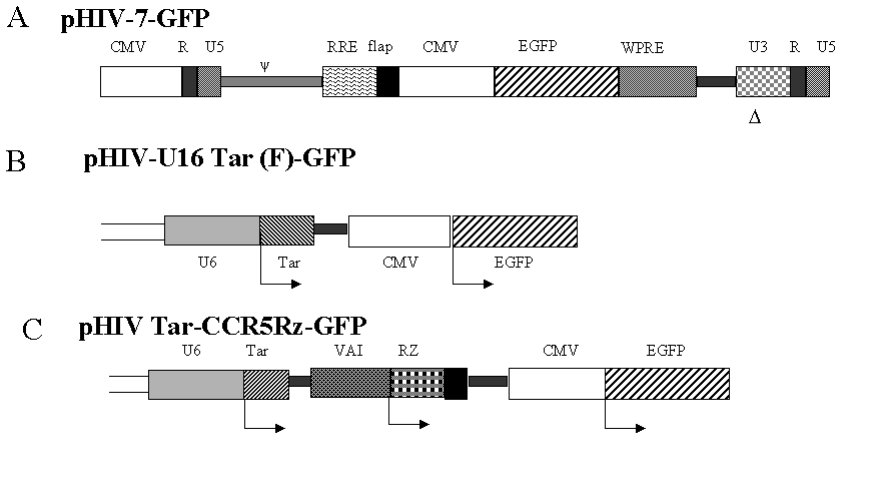
HIV-1 based lentivirus transfer vectors: A, control vector pHIV-7-EGFP with an EGFP reporter gene driven by the CMV promoter. B, HIV-U16Tar (F)-GFP vector with U6 driven Tar decoy. C, HIV-Tar-CCR5 ribozyme vector with U6 driven Tar and VA1 driven CCR5 ribozyme [18]. To generate vector viruses, a four-plasmid transfection system was used as described in methods.

**Figure 2:**
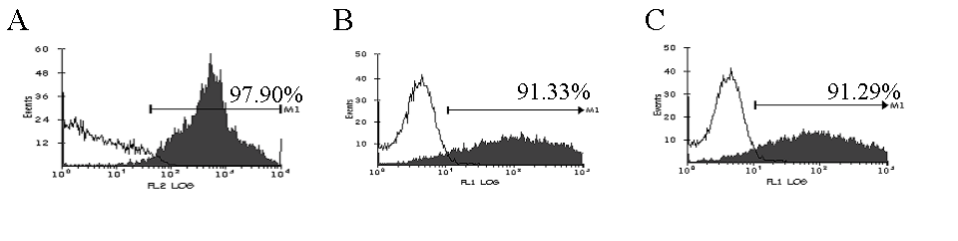
CD34+ cell purity and transduction efficiency: CD34+ cells derived from human fetal liver were purified by immunomagnetic beads and assayed by FACS. Percentage purity is indicated in A. Purified CD34+ cells were transduced with HIV-1 Tar (B) and Tar-CCR5 ribozyme (C) containing lentiviral vector. Percentages of EGFP positive cells at 48 hrs post-transduction are indicated in panel B and C. Isotype antibody control is shown in each panel (unshaded areas).

Early studies using MuLV based retrovirus vectors have shown the efficacy of aptamers and ribozymes against Tat, Rev, or envelope in interfering with HIV-1 infection [5,6,10,20]. Down regulation of CCR5 by the ribozyme used here and its corresponding inhibitory effect on HIV-1 infection was described previously [5,6,19]. To further expand the utility of such inhibitory RNAs, we used a third generation HIV-1 based self-inactivating vector (Fig. 1) [21] A highly enriched population of human CD34+ cells (>90% pure) were used for vector transductions. A representative FACS profile of purified CD34+ cells is shown in Fig. 2A. Transduction efficiency, as determined by FACS for EGFP at 48 hrs post-transduction, showed high levels of gene transfer and exceeded 90% for both U16Tar decoy and Tar-CCR5 vector constructs (Fig. 2, panel B & C).

### Tar and Tar-CCR5Rz vector transduced CD34+ cells differentiate normally into mature macrophages

**Figure 3:**
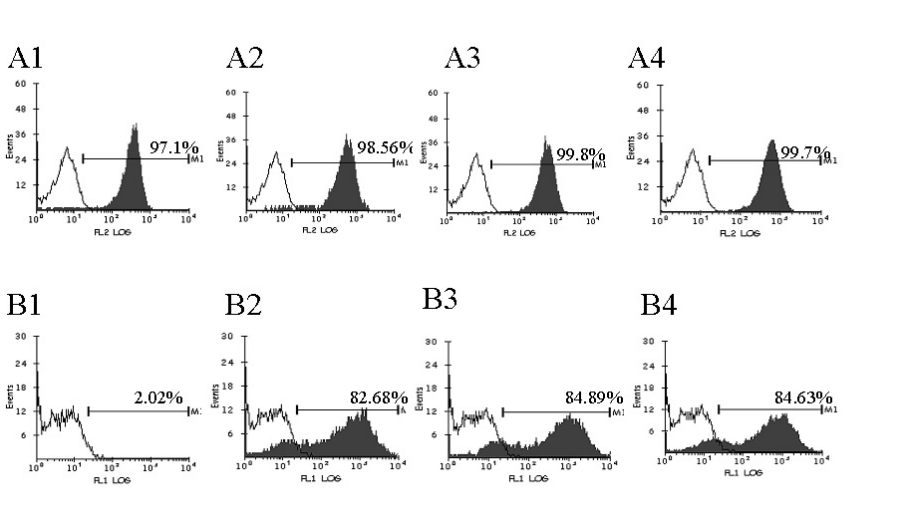
FACS analysis of transgenic macrophages for the CD14 surface marker and EGFP expression: Control and HIV-1 Tar & Tar-CCR5Rz vector transduced CD34+ cells were differentiated into macrophages *in vitro *in cytokine medium. Cells were stained for the macrophage surface marker CD14 using CD14-PE antibodies. Cells were analyzed by FACS for CD14 and EGFP. Panel A: CD14 staining of macrophages. 1, Nontransduced cells; 2, Control EGFP vector transduced; 3, HIV-1-Tar vector transduced; 4, HIV-1-Tar-CCR5Rz transduced. Percent CD14 positive cells are indicated together with isotype staining controls (unshaded areas). Panel B: EGFP expression by transduced macrophages. 1, Nontransduced cells; 2, Control EGFP vector transduced; 3, HIV-1-Tar vector transduced; 4, HIV-1-Tar-CCR5Rz vector transduced. Percent EGFP positive macrophages are indicated.

**Figure 4:**
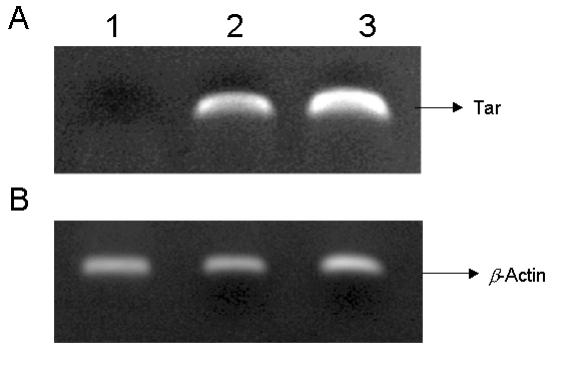
RT-PCR detection of Tar RNA in differentiated macrophages: Total cellular RNA was extracted from control and transgenic macrophages and subjected to RT-PCR using primers specific for HIV-1 Tar decoy. A. HIV-1 Tar specific amplification (125 bp). 1, control macrophages; 2, HIV-1-Tar vector transduced macrophages; 3, HIV-1-Tar-CCR5Rz vector transduced macrophages. B. *β*-actin RNA amplified in corresponding lanes as controls.

It is not known if lentivirus transduced Tar decoy and Tar-CCR5Rz, will have any adverse effects on the lineage specific differentiation of CD34+ cells into different end stage cells. Our results showed that both control and vector transduced cells matured normally into erythroid and myeloid colonies and no significant differences were observed between their colony forming abilities (data not shown). To determine if Tar and Tar-CCR5Rz RNA expressing CD34+ cells can give rise to mature macrophages, myeloid colonies were pooled and allowed to differentiate into adherent cells in cultures supplemented with M-CSF and GM-CSF for a period of 7 days. Results showed that cells derived from control, vector alone, or vector expressing transgenes (Tar and Tar-CCR5Rz) showed similar pattern of CD14 expression (Fig. 3, panel A1 to A4). The transgenic macrophages were also analyzed for the levels of EGFP reporter expression. As expected, the nontransduced cells did not show any EGFP expression (Fig. 3, panel B1) but cells transduced with either EGFP vector alone (panel B2) or vector with transgenes (panel B3 and B4) were strongly positive (>80%) for EGFP production. RT-PCR analysis confirmed the expression of the transgene Tar. Tar specific products of expected size (125 bp) were detected in both Tar (Fig. 4, panel A, lane 2) and Tar-CCR5Rz (lane 3) vector transduced macrophages. Control nontransduced macrophages, as expected, did not show any specific product (lane 1). No difference in the amounts of control *β*-actin RNA could be seen in the corresponding lanes (Fig. 4, panel B).

### Tar and Tar-CCR5Rz transgenic macrophages resist HIV-1 challenge

**Figure 5:**
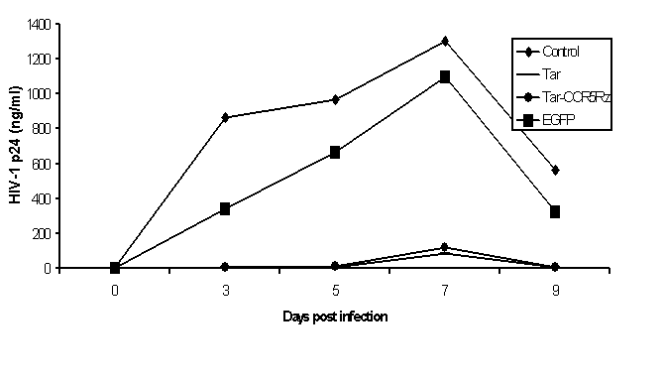
HIV-1 challenge of differentiated macrophages: HIV-1 Tar, Tar-CCR5Rz and control EGFP vector transduced and unmanipulated control CD34+ cells were allowed to differentiate into macrophages *in vitro*. Later, they were challenged with a macrophage tropic HIV-1 strain BaL. Viral supernatants were collected at different times post-infection and assayed for p24 antigen by ELISA. Values represent averages of duplicate cultures.

To determine if Tar and Tar-CCR5Rz transduced *in vitro *differentiated macrophages resist HIV-1 challenge, they were infected with R5-tropic HIV-1 Bal strain. Culture supernatants collected on different days post challenge were assayed for p24 antigen by ELISA. Compared to unmanipulated control or EGFP control, both Tar and Tar-CCR5Rz expressing macrophages showed remarkable resistance against HIV-1 challenge (Fig. 5). Small amounts of p24 could be detected on day seven and none at nine days post infection in Tar and Tar-CCR5Rz transduced cells.

### Tar and Tar-CCR5Rz transduced CD34+ cells can give rise to thymocytes in SCID-hu thy/liv grafts

**Figure 6:**
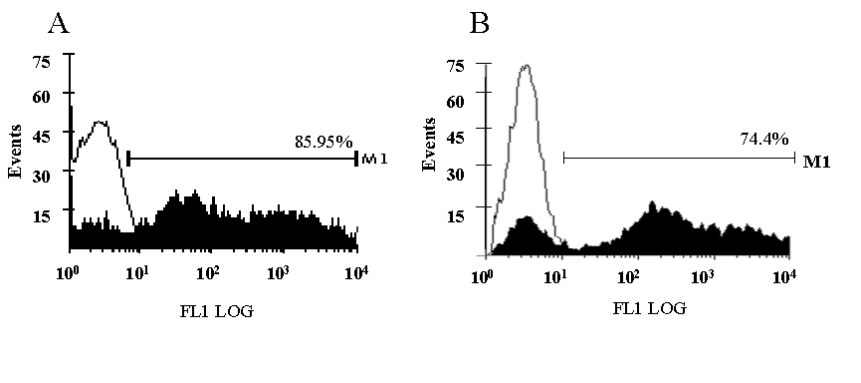
EGFP expression by in vivo derived thymocytes: HIV-1-Tar and Tar-CCR5Rz vector transduced CD34+ cells were injected into the SCID-hu mice thy/liv grafts and allowed to differentiate into thymocytes. At ~60 days post-engraftment, the cells were harvested and analyzed by FACS for EGFP expression. A. thymocytes from HIV-1 Tar transduced cells. B. thymocytes from Tar-CCR5Rz transduced cells. Percent positive cells are indicated. Representative samples from one mouse each are shown.

**Figure 7:**
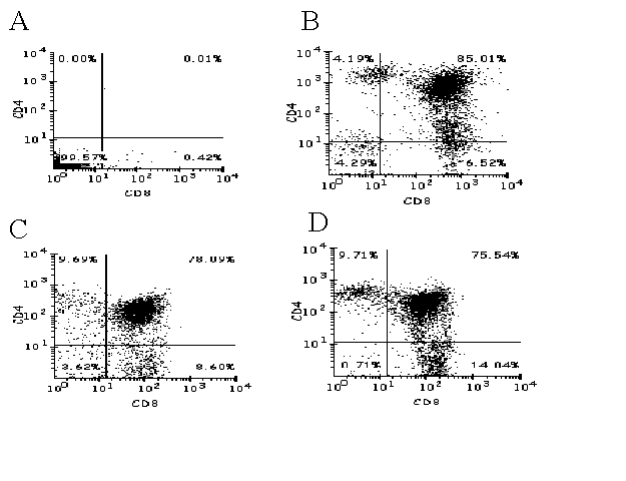
FACS profiles of thymocyte subsets derived in SCID-hu grafts: To determine the presence of different thymocyte subsets, *in vivo *differentiated cells from SCID-hu grafts were collected and stained for thymocyte markers CD4 and CD8 by using PE and FITC conjugated antibodies respectively. The stained cells were analyzed by two color FACS. A, Isotype control. B, thymocytes from a control animal; C, thymocytes from a HIV-1 Tar animal; D, thymocytes from a HIV-1-Tar-CCR5Rz animal.

Human thy/liv grafts in SCID-hu mice provide an ideal environment for CD34+ cells to mature into thymocytes. To determine if the lentivirally expressed transgenes Tar and Tar-CCR5Rz would have any adverse effect on this differentiation process, thymocytes obtained from SCID-hu grafts 60–70 days post reconstitution were analyzed for EGFP expression. All of the four mice (two each with Tar and Tar-CCR5Rz) that were injected with transduced CD34+ cells were positive for the presence EGFP expressing thymocytes. Of the two mice injected with Tar construct one showed 85% reconstitution levels with the other being 33%. For the two Tar-CCR5Rz construct injected mice, the reconstitution levels were 75% and 30% (Fig. 6, panels A and B). Reconstitution levels are known to vary considerably between mouse to mouse based on the variable sizes of the grafts injected and possibly due to the varying numbers of true stem cells present in the samples injected [22,23]. Since vector transduced CD34+ cells gave rise to EGFP expressing thymocytes in SCID-hu grafts, these results suggested that expression of either Tar or Tar-CCR5Rz RNA did not have any detectable deleterious effects on the thymopoiesis steps *in vivo*. FACS analysis was carried out on biopsied thymocytes by staining for CD4 and CD8 antigens to evaluate the presence of different cell subsets. The majority of the thymocytes (75 – 80%) stained positive for CD4 and CD8 (double positive) consistent with normal thymopoiesis (Fig. 7, panels B to D). Similar to the control (B), both CD4 and CD8 single positive mature thymocytes are also seen in Tar and Tar-CCR5Rz transduced thymocytes derived in SCID-hu grafts (C and D). These data indicated normal development of all three thymocyte subpopulations from Tar and Tar-CCR5Rz transduced CD34+ cells. When these cells were cultured *in vitro *for an additional 7 days, a rapid decline in the number of CD4/CD8 double positive cells was observed which coincided with a corresponding increase in single positive mature thymocytes (data not shown). To determine if the transgenic thymocytes derived in the SCID-hu mice retained their ability for nonspecific mitogenic stimulation in the presence of IL-2, they were cultured in the presence of PHA-P for 3 days. Approximately, a 3-fold increase in the number of thymocytes was observed for both control as well as transduced cells (data not shown). These cells expressed the chemokine receptor CXCR4, as expected (data not shown).

### *In vivo *derived transgenic thymocytes resist HIV-1 challenge

**Figure 8:**
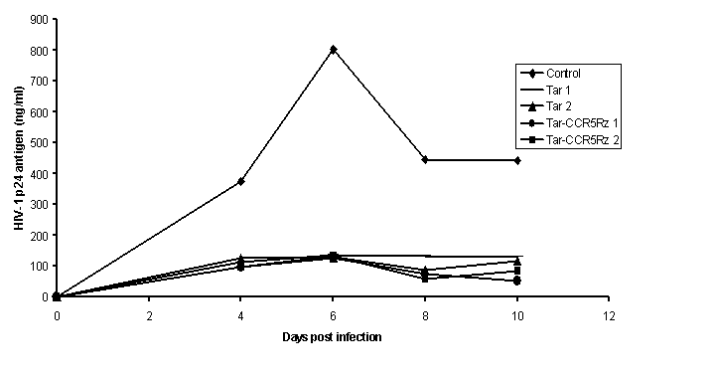
HIV-1 challenge of *in vivo *differentiated thymocytes: Vector transduced CD34+ cells were injected into SCID-hu thymic grafts and allowed to differentiate. Thymocytes were harvested from two different mice at 60 days post-engraftment. To enrich for the EGFP positive cells, they were sorted by FACS (>90% purity). The cells were expanded by culturing *in vitro *and challenged with the T cell tropic HIV-1 NL4-3. On different days post-infection, samples were collected and assayed for p24 antigen. Tar 1 & 2 and Tar-CCR5Rz 1 & 2 represent data from two different mice.

To determine if Tar and Tar-CCR5Rz RNA expressing thymocytes display resistance to HIV-1 replication, FACS sorted EGFP positive cells were challenged with a T-tropic HIV-1 NL4.3 virus *in vitro *(Fig. 8). Thymocytes isolated from both groups of mice (Tar and Tar-CCR5Rz transduced) showed remarkable resistance to HIV-1. In contrast, control unmanipulated thymocytes produced large amounts of virus (~8 fold higher p24 antigen on day 6). These control cells continued to produce detectable virus until 25 days post infection (data not shown)

## Discussion

The future success of stem cell based gene therapy strategies against HIV-1 infection depends on harnessing novel interfering genes for *in vivo *application in humans. This requires collective utilization of stem cell gene transduction with novel vectors followed by preclinical evaluation of gene therapeutic constructs in an *in vivo *setting. To achieve this goal, we used lentiviral vectors to transduce CD34+ cells with a Tar decoy alone or in combination with an anti-CCR5 ribozyme that down regulates an essential HIV-1 coreceptor. Tar decoy interferes with an essential HIV-1 regulatory gene thus inhibiting post-entry steps of viral replication, whereas the anti-CCR5 ribozyme helps prevent viral entry. In view of the previously demonstrated *in vitro *high efficacy of aptamers targeted to different HIV-1 proteins and the high likelihood of these being exploited for clinical use, it is essential that they be tested thoroughly *in vivo*. These experiments mark the first simultaneous evaluation of these constructs in lentivirally transduced CD34+ cells both *in vitro *and *in vivo*.

The use of lentiviral vectors permitted higher levels of gene transfer (>90%) into CD34+ cells with both of the constructs as assayed by EGFP expression. Two rounds of transduction with a highly concentrated VSV-G pseudotyped vector helped achieve high levels of gene transfer. Cells continued to express EGFP throughout the experimental period when cultured *in vitro *in the presence of cytokines and growth factors, and as long as 70 days *in vivo *in SCID-hu mice. The self-inactivating lentiviral vector employed here incorporated two important *cis *elements, namely a flap region and a WPRE element, to achieve high levels of EGFP expression [21]. Additionally, to minimize promoter interference, EGFP reporter, Tar, and CCR5 ribozyme genes were placed under the control of three different promoters namely, CMV, U6 and VA1 respectively [18]. Based on the high levels of gene transfer and sustained expression obtained, lentiviral vectors are highly suitable for gene transfer of RNA decoys and ribozymes.

CD34+ cells can be differentiated into myeloid, erythroid, and macrophage cell progeny in the presence of appropriate growth factors *in vitro*, and into mature T lymphocytes *in vivo *in SCID-hu mice [6,23]. *In vitro *CFU assays yielded similar levels of differentiated erythroid and myeloid colonies, and in long term culture with cytokines, more than 90% of cells matured into macrophages and expressed normal levels of CD14. Remarkably, EGFP production also remained very high (>80%) in transgenic macrophages. Thus lentivirus mediated Tar and Tar-CCR5Rz transgene expression did not adversely interfere with the differentiation of CD34+ cells into different lineages including macrophages. In *in vivo *experiments with SCID-hu mice, cell biopsies analyzed 60 to 70 days post engraftment showed that both Tar and Tar-CCR5Rz transduced progenitor cells matured into T-lymphocytes. During the normal course of thymopoiesis, the T cell precursors initially give rise to CD4 and CD8 double positive immature cells followed by subsequent end stage maturation into single positive CD4 and CD8 cells [24]. It is possible that transgene expression may selectively alter maturation of different cell subsets. Our results showed the presence of all three thymocyte subsets in grafts reconstituted with transduced cells when compared to control cells. Additionally, when the transgenic thymocytes were sorted and cultured *in vitro*, the levels of immature thymocytes declined rapidly with a corresponding increase in single positive CD4 and CD8 cells demonstrating their capacity to mature. Collectively, this data established that transduced CD34+ progenitor cells can differentiate normally into mature macrophages and thymocytes thus indicating no apparent toxicity of these constructs on lineage specific differentiation.

Viral challenge experiments demonstrated that both Tar and Tar-CCR5Rz RNA expressing mature T-lymphocytes and macrophages are remarkably resistant to HIV-1 infection. No synergistic effect could be observed with the combinatorial construct most likely due to the predominant effect of the Tar decoy itself at the low m.o.i. used here. However, synergistic effect of the combinatorial construct was demonstrated in previous studies that used a higher challenge dose [18]. In early studies with similar constructs using MuLV based retrovirus vectors, [6,20] viral inhibition was seen up to 2 weeks post challenge in differentiated thymocytes. However, there was a significant viral breakthrough by the third week. In contrast, with the lentiviral vector delivered constructs employed here, virus production in both challenged macrophages and T-lymphocytes remained significantly lower throughout the three week observation period. This improved level of protection is likely due to higher levels of gene transduction and expression, lower levels of transgene silencing during cell differentiation steps, or a combination of both. Although the levels of viral inhibition achieved in transgenic macrophages and T-lymphocytes are highly significant, small amounts of viral production is still detectable. This could be due to sub-optimal levels of transgene expression in a subpopulation of cells, or alternatively due to the presence of a small number of non-transduced cells in culture. Nevertheless, the above results demonstrated the efficacy of these transgenes in a combinatorial setting in a stem cell-based gene therapy context. The above results paved the way for exploiting this approach in human clinical trials.

## Conclusions

High efficiency transduction and sustained expression of HIV-1 interfering genes, anti-CCR5 ribozyme and HIV-1 Tar aptamer, could be achieved in CD34+ hematopoietic progenitor cells by using lentiviral vectors. The transduced progenitor cells differentiated normally into mature thymocytes *in vivo *in thy/liv grafts of SCID-hu mice and into normal macrophages *in vitro*. When challenged with HIV-1, transgenic cells showed marked resistance against HIV-1 infection. These results showed for the first time that expression of these transgenes in combination do not interfere with normal thymopoiesis and thus have set the stage for their application in stem cell based gene therapy for HIV/AIDS.

## Methods

### Tar decoy and Tar-CCR5Rz containing lentiviral vectors

The design, structure, and *in vitro *efficacy in cultured cells of anti-CCR5 ribozyme, Tar decoy, and Tar-CCR5Rz constructs were described previously [5,10,18,19]. These inhibitory RNAs introduced into a third generation self-inactivating lentiviral vector were used in the present study [21]. The transfer vector pHIV-7-GFP containing a CMV driven EGFP reporter gene is depicted in Fig. 1, panel 1A. Two important unique features are the *cis*-acting elements, the HIV-1 central flap sequence and the woodchuck post-transcriptional regulatory element (WPRE) for optimal EGFP expression. In the transfer vector pHIV-U16-Tar-GFP, the Tar decoy under the control of the U6 promoter was positioned upstream of the EGFP reporter (Fig 1B). In the combinatorial construct pHIV-U16Tar-CCR5Rz-GFP, the Tar decoy is driven by U6 whereas the CCR5 ribozyme is under the control of the VA1 promoter (Fig. 1C).

### Production of high titered retroviral vectors

To generate vector stocks, 293T cells were transfected with 15 μg of pCHGP-2 (encodes HIV-1 gag/pol), 15 μg of transfer vector (pHIV-7 GFP or pHIV-U16 Tar-GFP or pHIV-U16Tar-CCR5Rz-GFP), 5 μg of pCMV-rev and pCMV-G each as described previously [23]. Viral supernatants were collected at 24, 48 and, 72 hrs post transfection, pooled, and concentrated by ultracentrifugation [25]. Concentrated virus was resuspended in a small volume (500 μl) of DMEM containing 10% fetal bovine serum. The titer of the vector preparation was determined in 293T cells as described previously and ranged from 1 to 3 × 10^8 ^TU/ml. Multiple aliquots were made and stored at -70°C.

### Isolation of CD34+ hematopoietic progenitor cells and high efficiency vector transduction

Human fetal liver CD34+ hematopoietic progenitor cells (HPC) were purified by positive selection on a magnetic column using the Direct CD34 Progenitor Cell Isolation Kit from Miltenyi Biotech, Gladbach, Germany, as described in detail earlier [23]. Purified cells were suspended in Iscove's medium supplemented with IL3, IL6, and human stem cell factor (SCF), each at a concentration of 100 ng/ml (R & D Systems, Minneapolis, MN) and cultured for 15 hours at 37°C. Vector transductions were carried out in a 12-well tissue culture plate using 2 × 10^6 ^cells at an m.o.i. of 10 to 20 in a final volume of 100 μl of medium containing 4 μg/ml polybrene. Following transduction, cell aliquots were used for carrying out *in vitro *colony forming unit (CFU) assays, generation of macrophages, and for reconstitution of human thy/liv grafts in SCID-hu mice to generate T cells.

### CFU assays and generation of macrophages

Control, or vector transduced CD34+ cells were allowed to differentiate into multiple lineages of erythroid and myeloid lineages in a semi-solid medium (MethocultTM GF H4434, Stem cell Technologies, Vancouver, BC, Canada). This medium contains the following components: 1% Methylcellulose in Iscove's MDM, 30% fetal bovine serum, 1% bovine serum albumnin, 0.1 mM 2-mercaptoethanol, 2 mM glutamine, 50 ng/ml rh stem cell factor, 10 ng/ml rh GM-CSF, 10 ng/ml rh IL-3, and 3 units/ml rh erythropoietin. A colony forming unit (CFU) was defined as having at least 50 cells after 14 days in the above selective medium. Individual myelomonocytic colonies were pooled and cultured in DMEM supplemented with 10% fetal bovine serum, 50 ng/ml M-CSF, and 20 ng/ml GM-CSF, for a period of 14 days for differentiation into macrophages. Cells were stained for CD14 antigen and analyzed by FACS to determine macrophage yield.

### Reconstitution of SCID-hu grafts with transduced CD34+ cells and derivation of T cells

Human fetal thymus and liver tissues were implanted under the kidney capsule of SCID mice to generate SCID-hu mice as described earlier [26]. Control and lentivirus vector transduced CD34+ progenitor cells (1 × 10^6^) were injected directly into thy/liv grafts for reconstitution. Eight to ten weeks post reconstitution, thus allowing for T cell differentiation, the animals were sacrificed and thymocytes were isolated from the grafts. The differentiated thymocytes were cultured *in vitro *and checked for their ability to respond to mitogen, PHA-P and interleukin-2. Briefly, thymocytes were washed once with medium containing serum and resuspended into Iscove's medium supplemented with 10% fetal bovine serum. Approximately 2 × 10^6 ^cells were plated in a 12 well tissue culture plate and stimulated with PHA-P (4 μg/ml) and IL-2 (10 U/ml) for three days. Cells were counted after 3 days to determine expansion.

### PCR Detection of Tar RNA

Total RNA was isolated from approximately 1 × 10^6 ^control and transgenic macrophages using Qiagen RNA/DNA mini kit (Qiagen, Germany) and subjected to RT-PCR as described before [20]. In each case, a 125 bp DNA fragment is expected. The following primers were used: 1, Forward Tar: 5'-GCAATGATGTCGTAATTTGC and 2, Reverse Tar: 5'-CTTGCTCAGTAAGAATTTTCGTC.

### HIV-1 infection of thymocytes

Thymocytes derived from thy/liv grafts of SCID-hu mice were sorted by FACS to enrich for EGFP expressing cells (>90% purity). They were later expanded by stimulation by PHA-P in medium containing serum and IL-2 as described earlier [6,23]. Approximately 10^6 ^cells were infected with HIV-1 NL4-3 at an m.o.i. of 0.001 in a final volume of 100 μl for 3 hrs at 37°C. Infected cells were washed twice with DMEM with 10% fetal bovine serum and cultured in a 12 well plate for 3 weeks. Supernatants (0.5 ml) were collected on alternative days with media replenished in each well. Amounts of virus produced in cell culture supernatants was measured by HIV-1 p24 ELISA.

### HIV-1 challenge of CD34+ cell derived macrophages

Infection with a macrophage-tropic Bal-1 strain of HIV-1 was carried out in a 6 well plate. Approximately 2 × 10^6 ^adherent macrophages differentiated *in vitro *were infected with Bal-1 virus at an m.o.i. of 0.001 in the presence of 4 μg/ml of polybrene for 6 hours. Thereafter, 3 ml of DMEM supplemented with 10% serum was added. Supernatants (0.5 ml) were collected every other day from each well for 3 weeks and stored at -70°C. The levels of virus released were determined by p24 antigen ELISA.

## Competing interests

The author(s) declare that they have no competing interests.

## Author's contributions

AB carried out most of the experiments. M Li and JR were responsible for vector design and preparation. LR assisted in SCID-hu mice generation, CD34 cell reconstitutions into mice, PCR and FACS analysis. RA was responsible for the overall experimental design and implementation of the project.
